# Ionisation bias undermines the use of matrix‐assisted laser desorption/ionisation for estimating peptide deamidation: Synthetic peptide studies demonstrate electrospray ionisation gives more reliable response ratios

**DOI:** 10.1002/rcm.8441

**Published:** 2019-05-10

**Authors:** Joanna P. Simpson, Martin Fascione, Ed Bergström, Julie Wilson, Matthew J. Collins, Kirsty E.H. Penkman, Jane Thomas‐Oates

**Affiliations:** ^1^ Department of Chemistry University of York York UK; ^2^ Centre of Excellence in Mass Spectrometry University of York York UK; ^3^ Department of Archaeology University of York York UK; ^4^ Department of Mathematics University of York York UK

## Abstract

**Rationale:**

Although mass spectrometry (MS) is routinely used to determine deamination in peptide mixtures, the effects of the choice of ionisation source have not yet been investigated. In particular, matrix‐assisted laser desorption/ionisation (MALDI) has become a popular tool with which to measure levels of glutamine deamidation in ancient proteins. Here we use model synthetic peptides to rigorously compare MALDI and electrospray ionisation (ESI).

**Methods:**

We used two synthetic peptides, with glutamine (Q) in one substituted for glutamic acid (E) in the other, to investigate the suitability of MALDI and ESI sources for the assessment of deamidation in peptides using MS. We also compared measurements of the same Q‐ and E‐containing peptide mixtures using two different mass analysers (time‐of‐flight (TOF) and Fourier transform ion cyclotron resonance (FT‐ICR)).

**Results:**

When standard mixtures of the Q‐ and E‐containing peptides were analysed using MALDI, under‐representation of the E‐containing peptide was observed. This observation was consistent between analyses carried out using either TOF or FT‐ICR‐MS. When the same mixtures were analysed using ESI FT‐ICR‐MS, no ionisation bias was observed.

**Conclusions:**

MALDI may not be a suitable ionisation method for the determination of deamidation in peptide mixtures. However, ESI was successfully used to determine the ratio in known mixtures of Q‐ and E‐containing peptides. These preliminary observations warrant further investigation into ionisation bias when measuring deamidation in other peptide sequences.

## 1 INTRODUCTION

Deamidation of proteins has been described as a molecular clock,^1^ with its rate dependent on a few known factors; such as the residue in which it occurs, with asparagine (N) found to be less stable than glutamine (Q).^2^ Deamidation measurements provide a versatile tool for the investigation of protein stability and diagenetic changes. It is therefore not surprising that deamidation has been used in a broad range of research areas, from modern medicinal applications such as those in biotherapeutics,^3^ to the investigation of deamidation products in relation to protein aging, such as α‐crystallin in eye proteins,^4^, ^5^ studies on the proteome of human hair,^6^ shotgun proteomic applications assessing deamidation in complex biological samples^7^ antibody and peptide therapies,^8^, ^9^ as well as more recent applications such as the use of hydrogen/deuterium exchange mass spectrometry (HDX‐MS) to investigate the effects of deamidation on monoclonal antibody structural confirmations.^10^

In addition to this there have been a wide range of archaeological applications, with proteins recovered from ancient artefacts from sites as old as 3.8 million years in age.^11^ This has resulted in a wide range of sample types being reported in the literature, including: keratin in wool,^12^, ^13^ keratin in mummified skin,^14^ collagen in bone,^15^, ^16^ and protein binders in paint.^17^ Understanding the mechanisms and conditions that drive this reaction is important when trying to measure and quantify levels of deamidation. A number of studies in the literature have used analytical techniques such as liquid chromatography (LC) and, more recently, MS to measure levels of Q and/or N deamidation in biological^18^, ^19^ and synthetic peptides.^20^, ^21^ In order to determine the ratios of glutamine to glutamic acid (E) using MS, it has generally been assumed by researchers that each of the two forms of the peptide (deamidated and undeamidated) have equal ionisation efficiencies. If this assumption is correct, the ratios of the peak intensities of the Q‐ and E‐containing species will be directly proportional to the concentration ratios of the two peptide forms.

Matrix‐assisted laser desorption/ionisation (MALDI) coupled to time‐of‐flight (TOF)‐MS has been routinely used for the analysis of peptide mixtures and, over recent years, for the estimation of levels of deamidation in various sample types.^12^, ^13^, ^16^, ^22^, ^23^ One disadvantage of this instrumentation is that due to the insufficient resolving power of the mass analyser, it is not possible to fully resolve the signals for the deamidated (+0.984 Da) and amidated peptide forms. For example, the mono‐isotopic signal of the deamidated (E‐containing) peptide overlaps with the signal for the undeamidated (Q‐containing) species. The deamidated mono‐isotope differs from the amidated second isotope by only 0.02 Da, with isotopic envelopes becoming more convoluted with increasing deamidation events. In order to calculate the Q/E ratio, a genetic algorithm has been used to deconvolute the overlapping isotopic distributions of the two peptide forms.^24^

Authentic peptide standards are required in order to investigate the accuracy of both the ionisation methods, and the efficiency of the algorithm^24^ for the estimation of Q or N deamidation in biological samples. Consequently, in this study two peptides have been synthesised with the amino acid sequences YAYGOGQVG and YAYGOGEVG, where O is used to denote hydroxyproline. The synthesised peptides were analysed using LC/MS to assess their purity and the two peptide forms were mixed in known ratios. Analysis was undertaken using MALDI‐TOF‐MS and MALDI‐FT‐ICR‐MS to determine levels of Q deamidation. Through this experiment, we therefore assess the applicability of MALDI for the estimation of Q/E ratios, and the efficiency of the previously published deconvolution method.^15^, ^24^ In addition, the mixtures were also analysed using the ESI source on the FT‐ICR instrument for direct comparison.

## 2 EXPERIMENTAL

### 2.1 Solid‐phase peptide synthesis

When designing the peptides, several factors were taken into consideration. The peptides needed to be greater than 800 Da in mass, so as not to fall in the same region of the spectrum as the CHCA MALDI matrix peaks. The length of the peptide is also important, as longer peptides have more chance of secondary structures developing during synthesis, as well as generally decreasing peptide purity due to an increase in the number of coupling steps. Therefore, to maximise peptide purity, the peptide was chosen to be no longer than nine amino acids in length. The original context for this study was to investigate the measurement of deamidation in bioarchaeological studies (which commonly look at collagen‐derived peptides). However, we acknowledge that the study of deamidation in biological samples is a much wider question. These factors were taken into account when choosing the amino acid composition of the synthetic peptide. Similarities between the peptide presented here and a typical collagen sequence are, the inclusion of a hydroxyproline residue, and a third of the amino acid composition is glycine (G). As glycine is the smallest amino acid with a residue mass of 57 Da, two tyrosine residues (Y, residue mass of 163 Da) were included in the peptide to offset the small size of the glycine. Tyrosine was also useful as it contains a UV chromophore, enabling the peptides to conveniently be detected and quantified using high‐performance liquid chromatography (HPLC) with ultraviolet (UV) detection. The masses of the product peptides, YAYGOGQVG and YAYGOGEVG, are 926 Da and 927 Da, respectively. The details of the peptide synthesis are provided in supplementary note S1 (supporting information).

### 2.2 Measuring levels of deamidation using MS

This study used two mass spectrometers: a Fourier transform ion cyclotron resonance (FT‐ICR) mass spectrometer and a time‐of‐flight (TOF) mass spectrometer to generate spectra of the two product peptides (YAYGOGQVG and YAYGOGEVG) combined in various known proportions. These two synthetic peptides are referred to throughout as product peptide Q and product peptide E, respectively. The FT‐ICR mass spectrometer is a high‐resolution instrument and was fitted with MALDI and ESI sources. The second instrument is a lower resolution TOF instrument, with a fixed MALDI source. When using the FT‐ICR instrument it is possible to resolve the two peptide forms, as the *n* ^th^ peak of the deamidated peptide signal is resolved from the (*n* + 1)^th^ isotopic peak of the undeamidated signal. The overlapping isotopic distributions in the lower resolution MALDI‐TOF‐MS data were deconvoluted using the algorithm described in Wilson et al.^24^

### 2.3 Peptide purity analysis by HPLC/MS and MALDI‐TOF‐MS

For analysis using HPLC aliquots of the lyophilised peptides were re‐suspended in water at a concentration of 10 ppm. Each peptide was analysed using an HPLC‐HCTultra PTM Discovery System (Bruker Daltonics) fitted with a symmetry C18 3.5 μm column (4.6 × 7.5 mm; Waters) using mobile phases of acetonitrile (A) and water (B). The elution was isocratic with a flow rate of 1 mL/min and a total run time of 9 min.

For analysis by MALDI‐MS or ESI‐MS the peptides were prepared as follows. 2.02 mg of product peptide Q and 1.96 mg of product peptide E were transferred into separate plastic PP microfuge tubes and dissolved in 2 mL of a 50:50 mixture of ACN and purified water. The resulting 1000 ppm stock solutions were diluted to 10 ppm and mixed in the following weight ratios of Q/E: 100/0, 90/10, 70/30, 50/50, 30/70, 10/90 and 0/100. These mixtures were then used as calibration standards.

### 2.4 Measuring the ratios of the product peptides Q and E using MALDI‐TOF, MALDI‐FT‐ICR and ESI‐FT‐ICR

Once the purity of each peptide had been determined, the set of seven‐point calibration standards was then analysed using two different mass spectrometers and two ionisation sources. The two instruments used were the Bruker ultraflex III (TOF) and the Bruker solariX XR 9.4 T (FT‐ICR). The ultraflex was used with its fixed MALDI source in positive ion mode with 800 laser shots per sample acquisition. The data were acquired using flexcontrol software version 3.0 (Bruker Daltonics). Each spot was analysed in reflector mode using a smartbeam™ Nd:YAG laser (355 nm). Spectra were analysed using flexAnalysis software version 3.0 (Bruker Daltonics). The solariX was operated with either an ESI (direct infusion) or MALDI source in positive ion mode (800 laser shots per sample acquisition) with a smartbeam™ Nd:YAG laser (355 nm). Spectra were acquired using the solariXcontrol software and processed with DataAnalysis version 4.2 (Bruker Daltonics). Each sample was analysed in triplicate and the average values of the peak intensities are reported.

## 3 RESULTS

### 3.1 Purity analysis of peptides by LC/MS and MALDI‐TOF‐MS

#### 3.1.1 Demonstration of peptide sequences

Product ion analysis of the spots prepared with either 100% product peptide E or product peptide Q was carried out on the MALDI‐TOF/TOF instrument. The assignment of the sequence of each of the peptides was made on interpretation of the product ion spectra. The product ion spectrum for product peptide E showed a mixture of both the Q‐ and E‐containing peptides (the precursor selection window on the instrument used is >1 *m/z* unit). Harsh acidic treatments were used to cleave the synthetic peptides from the resin; it is therefore possible that some of the glutamine in the product peptide Q may have undergone minor deamidation during synthesis.

#### 3.1.2 Analysis of the purity of product peptides Q and E

**Figure 1:**
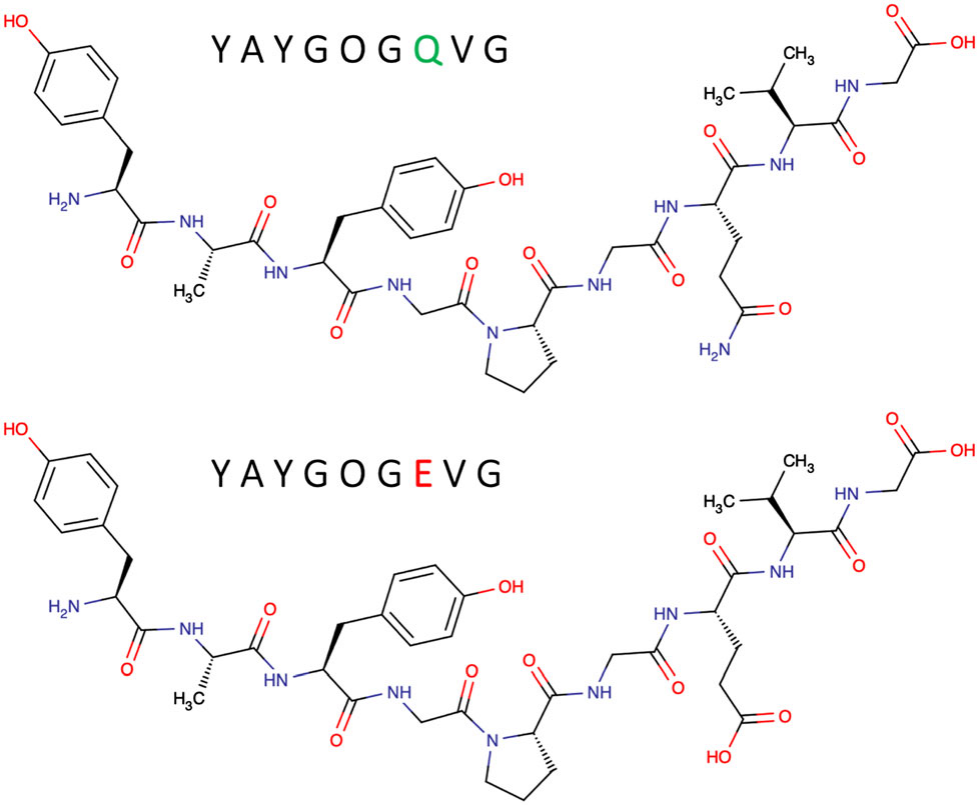
The structures of product peptides Q and E. Product peptide Q (top) has the amino acid sequence YAYGOGQVG with a mass of 926 Da. Product peptide E has the amino acid sequence YAYGOGEVG and a mass of 927 Da [Color figure can be viewed at wileyonlinelibrary.com]

To assess the purity of the two synthesised peptides, each product was analysed separately by reversed‐phase LC/ESI‐MS. UV absorbance (210–380 nm) data were also collected. As expected, similar absorbance patterns were observed for each of the two peptides. Using this LC/MS approach the product peptides Q and E were resolved chromatographically. Details are shown in supplementary note S2 (supporting information). On the basis of the peak areas observed in the UV chromatogram, product peptide Q is estimated to be ~95% pure and to contain 4% of peptide E, assuming that all components give a similar UV response. From this analysis it is not possible to distinguish whether the small amount of product peptide E in product peptide Q arises from deamidation during peptide synthesis, or from the presence of glutamic acid with the glutamine precursor used to make the peptide. Product peptide E was estimated to be ~95% pure and to contain ~3% of product peptide Q. The structures of product peptides Q and E are shown in Figure 1 with the corresponding amino acid sequences.

### 3.2 Comparison of the ratio of product peptide Q to product peptide E signals, measured using different mass spectrometers and ionisation sources

To investigate possible differences in ionisation efficiency of the product peptides, the seven samples of differing Q/E ratios (Table 1) were analysed directly, without chromatographic separation, using both ESI‐FT‐ICR‐MS and MALDI‐FT‐ICR‐MS, making possible direct comparison of the two sets of results. In addition, the seven samples were also analysed using MALDI‐TOF‐MS. This analysis was carried out in order to compare the estimated ratios of Q/E‐containing peptides obtained using the deconvolution method described in Wilson et al^24^ with those results from the FT‐ICR experiments in which the different peptides are resolved.

**Table 1:** Corrected percentages of Q‐ and E‐containing peptides calculated using purity obtained from LC/MS analysis

Theoretical % of Q	Theoretical % of E	Corrected % of Q taking into account peptide purity	Corrected % of E taking into account peptide purity
0	100	3	97
10	90	12	88
30	70	31	69
50	50	50	50
70	30	69	31
90	10	87	13
100	0	96	4

#### 3.2.1 FT‐ICR‐MS results

**Figure 2:**
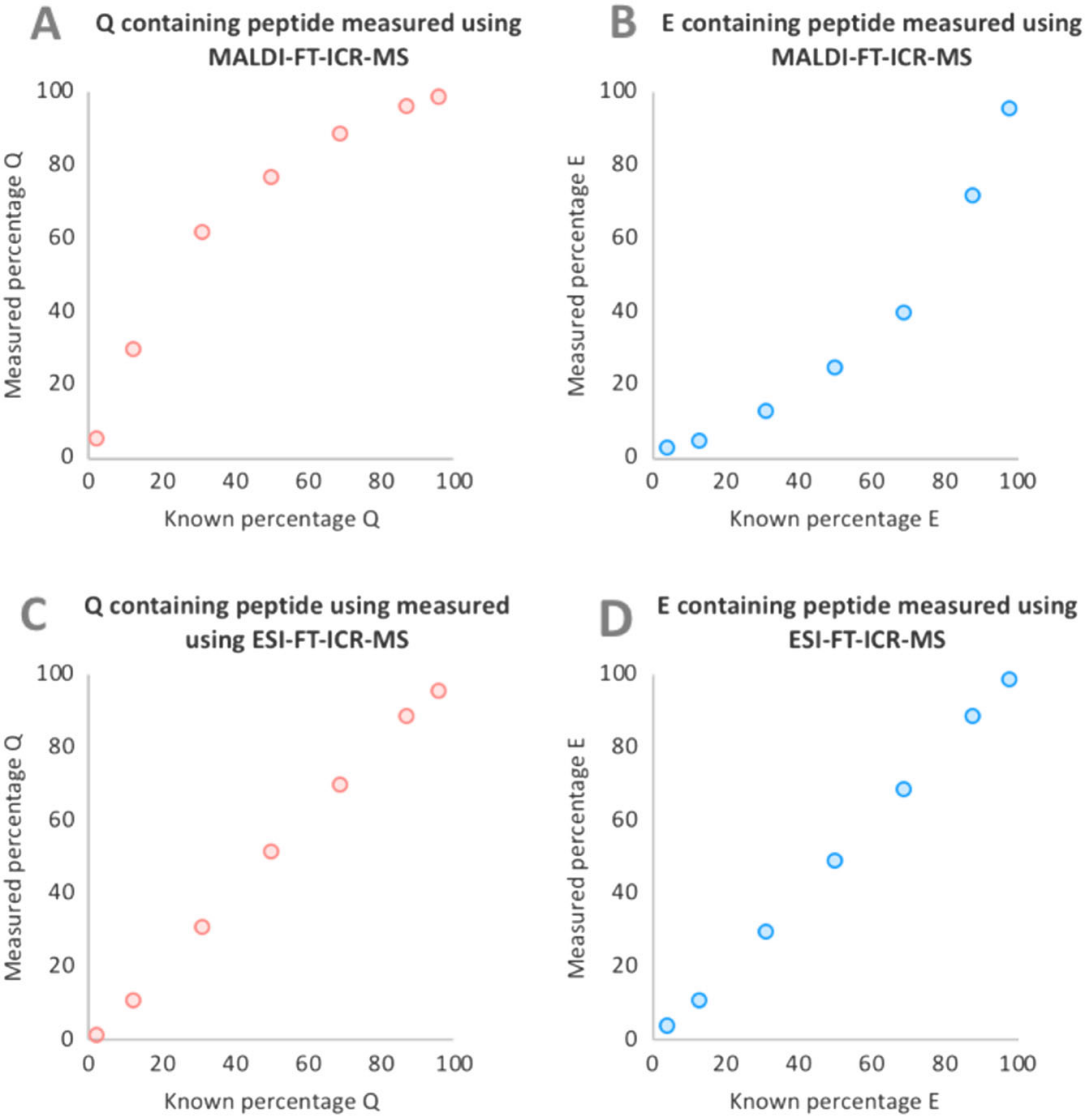
Plots showing measured percentage of product peptides Q and E, determined using MALDI (A and B) and ESI (C and D), against the known percentage. When using MALDI there is an underestimation of product peptide E [Color figure can be viewed at wileyonlinelibrary.com]

The peak intensities of the first three Q‐ and E‐containing peptide peaks in the isotopic distributions were measured using the Bruker solariX XR 9.4 T FT‐ICR mass spectrometer for each mixture using the two different ionisation methods (MALDI and ESI). Each peptide mixture was analysed six times and the average peak intensity of either product peptide Q or E was plotted against their corresponding theoretical percentages (Figure 2).

When the peptide mixtures were analysed using MALDI (Figures 2A and 2B), product peptide Q consistently produced higher peak intensities than product peptide E, regardless of the percentage concentration of the peptide. When using the ESI source (Figures 2C and 2D) the percentage of the Q‐containing peptide was predicted well across the mixture range, with the average difference between predicted and measured values being ~1%. The percentage of peptide Q in the same mixtures determined using MALDI resulted in a greater difference between predicted and measured values, with an average difference of ~15%, and differences ranging from ~3 to 30%. The errors were greatest within the 10–70% Q range, with calculated values for percentage Q overestimated by ~17 to 30%. It is evident that during MALDI in positive ion mode, ionisation of the less acidic Q‐containing peptide is favoured.

#### 3.2.2 TOF‐MS results

**Figure 3:**
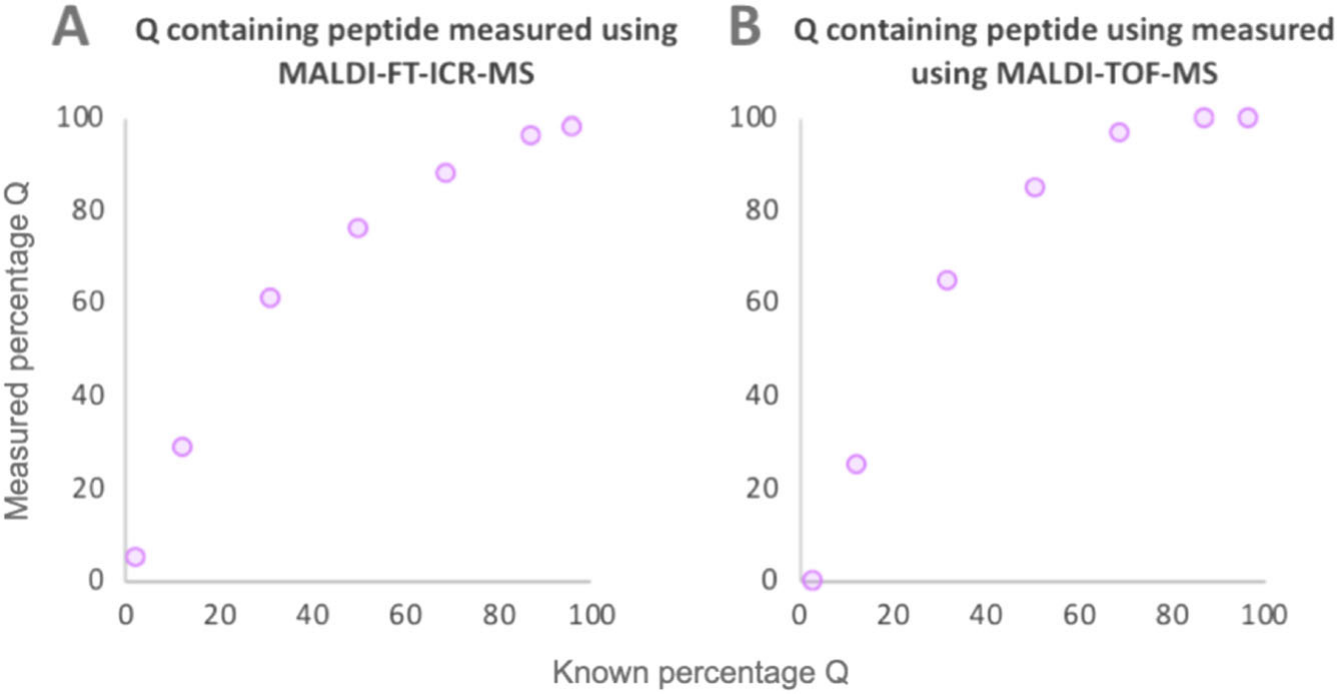
Ratios of Q‐ and E‐containing peptides, obtained using MALDI‐TOF‐MS (B). Due to an overestimation of the Q‐containing peptide a curvilinear relationship is observed. The same samples were analysed using a higher resolution FT‐ICR mass spectrometer (A), with the data showing a similar relationship [Color figure can be viewed at wileyonlinelibrary.com]

The peptide mixtures were also analysed using MALDI on a TOF mass spectrometer. The percentage of Q‐containing peptide in the seven peptide mixtures was estimated using the deconvolution method of Wilson et al^24^ (Figure 3). The differences between predicted and measured values obtained for the percentage of Q‐containing peptide ranged from ~3–35%, with the level of the product peptide E consistently underestimated. The results obtained using MALDI‐TOF‐MS showed a similar curvilinear relationship to that observed using MALDI on the FT‐ICR mass spectrometer. The algorithm of Wilson et al^24^ assumes equal ionisation efficiencies for Q‐ and E‐containing peptides and could perhaps be improved by taking ionisation bias into account. However, the study of multiple peptide sequences in a range of biological matrices would be required to investigate a possible correction factor.

#### 3.2.3 Investigating effects of peptide concentration, sample matrix and laser power on the obtained measurements of product peptides Q and E

As there appeared to be significant ionisation bias when measuring ratios of Q‐ and E‐containing peptides using MALDI, this was explored further. In addition to comparing ionisation sources we also investigated whether there were any effects caused by the concentrations of the peptide mixtures, the MALDI laser intensity, and the effects of the biological matrix. Known ratios of product peptides Q and E were spiked into three different tryptic protein digests: bovine serum albumin (BSA), cytochrome C (CytC) and collagen extracted from a bovine metatarsal bone, excavated from the Tanner Row site in 1994 by York Archaeological Trust. The bone is thought to date between the 11th and mid‐13th centuries (Tanner Row collagen extract).

**Figure 4:**
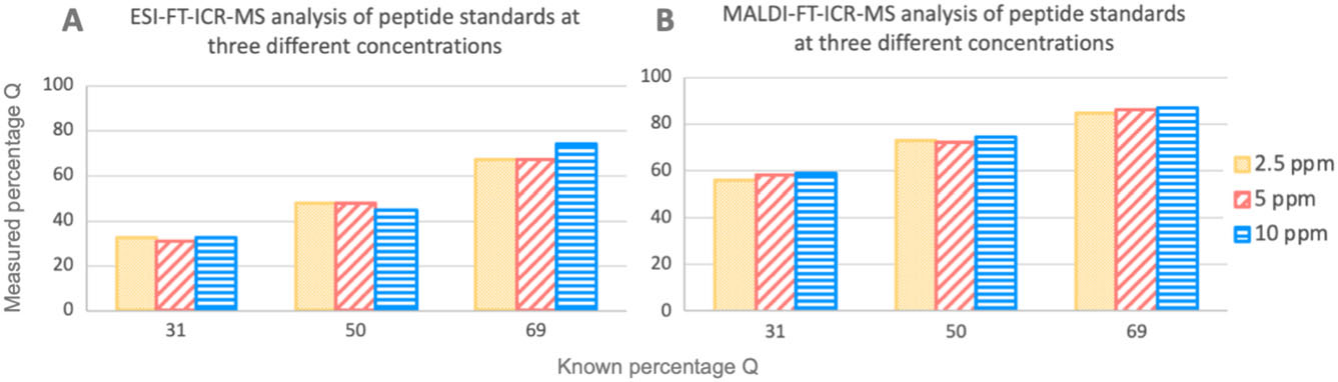
Measured percentage of Q in product peptide mixtures at three concentrations (2.5, 5 and 10 ppm). The data was obtained using either ESI‐FT‐ICR‐MS (A) or MALDI‐FT‐ICR‐MS (B) [Color figure can be viewed at wileyonlinelibrary.com]

In order to investigate the effects of concentration, the product peptide mixtures were analysed as standard mixtures in solvent, at three different final concentrations (2.5, 5 and 10 ppm). For each concentration three product peptide mixtures were analysed (30, 50 and 70% Q), using either ESI‐FT‐ICR‐MS or MALDI‐FT‐ICR‐MS (Figure 4).

When using ESI it was observed that the measured percentage Q across the three peptide ratios was consistent at concentrations of 2.5 and 5 ppm. For the mixture containing 50% product peptide Q, a measured percentage of 48 was obtained from both the 2.5 and 5 ppm solutions, while, from the 10 ppm solution, the measured level was slightly lower at 45%. For the mixture containing 70% product peptide Q, the same percentage Q (67%) was measured from both the 2.5 and 5 ppm solutions, with the 10 ppm solution resulting in a slightly higher measured value of 75%. When analysing the same mixtures using MALDI, the extent of overestimation of peptide Q slightly increases with the overall concentration of the peptide mixture.

**Figure 5:**
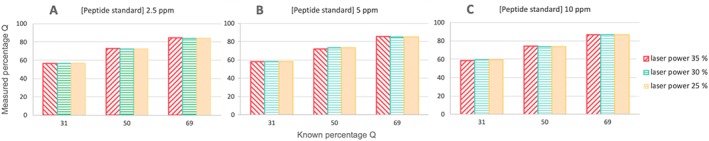
Measured percentage of Q in product peptide mixtures at three concentrations: (A) 2.5 ppm, (B) 5 ppm, and (C) 10 ppm analysed using three laser power percentages (35, 30 and 25%) [Color figure can be viewed at wileyonlinelibrary.com]

When using MALDI, one of the key instrument parameters that can be tuned is the percentage laser power used. The percentage laser power required to obtain good ionisation of a sample varies with sample type and instrument. As this is a key variable for MALDI analysis we tested the same product peptide mixtures (30, 50 and 70% Q) at three concentrations (2.5, 5 and 10 ppm), using three different laser powers (25, 30 and 35%) (Figure 5). We found that although the laser power used affected the quality of the resulting spectra, with a decrease in signal‐to‐noise (S/N) with decreasing laser power, it does not appear to significantly alter the measured levels of Q‐ and E‐containing peptides.

We have shown that there is an underestimation of product peptide E levels when using MALDI to analyse standard mixtures of two product peptides. However, biological samples such as protein tryptic digests are generally complex mixtures, which are likely to contain a number of Q‐ and E‐containing peptides. In order to test how accurately we can measure product peptides Q and E in such biological mixtures, standard peptide mixtures were spiked into tryptic digests of each of three proteins (bovine serum albumin (BSA), cytochrome C (CytC), and collagen extracted from an archaeological bovine bone). Each of the three digests was spiked with two different concentrations of product peptides (Table 2). Each sample was analysed by MS six times and, in each spectrum, the first three peaks in the isotopic distribution were summed and used to calculate the level of product peptides Q and E. An average of the percentages of Q and E derived from the six replicates was compared with the known percentage of Q for each sample.

**Table 2:** Measured percentages of Q in peptide mixtures spiked into tryptic protein digests (BSA, CytC and collagen) analysed using either ESI or MALDI

Biological matrix	[peptide spike] (ppm)	Known % Q	ESI		MALDI	
			Measured % Q	SD	Measured % Q	SD
BSA	5	31	34	2.98	58	2.21
BSA	10	31	34	1.63	60	0.83
BSA	5	50	64	3.63	74	0.42
BSA	10	50	56	0.59	74	0.42
BSA	5	69	85	1.41	85	1.28
BSA	10	69	77	1.65	87	0.73
CytC	5	31	29	0.29	56	3.00
CytC	10	31	31	3.78	61	0.46
CytC	5	50	47	2.21	57	1.13
CytC	10	50	45	0.56	61	0.46
CytC	5	69	65	0.61	88	0.63
CytC	10	69	65	2.27	88	0.29
Collagen	2.5	31	34	0.69	61	1.6
Collagen	5	31	32	1.26	61	3.04
Collagen	2.5	50	52	1.25	74	1.67
Collagen	5	50	52	1.03	75	0.91
Collagen	2.5	69	73	1.08	86	0.85
Collagen	5	69	70	0.38	86	0.48

SD: standard deviation

When analysed using ESI, peptide mixtures spiked into BSA tryptic digests showed the most variability between concentrations. Peptide mixtures spiked into CytC digests showed good reproducibility across the two concentrations, with ~4 and 5% underestimation of product peptide Q levels at 50 and 70% Q, respectively. Interestingly, given that collagen is the largest of the three proteins tested, when spiked into the archaeological collagen tryptic digests, the measurements of percentage Q showed the best consistency across the two concentrations. The measured percentage Q in collagen was accurate to within 4%. When the same spiked protein digests were analysed using MALDI there was a large underestimation of peptide E levels across all three sample types.

## 4 DISCUSSION

This study highlights the importance of investigating the suitability of analytical methodology to the question being asked: Including the suitability of the proposed mass spectrometric techniques used to answer research questions. The investigation of deamidation using mass spectrometry has been wildly applied and reported heavily in the literature; despite this, we are still discovering and learning, not only about the mechanism itself, but also about how the procedures we use during sample preparation and analysis can influence the resulting Q/E measurements, with papers published only recently detailing the effects of well‐established extraction procedures.^16^, ^25^ When deciding which ionisation source is most suitable for the samples in question, it is important to investigate relative mass spectrometric responses. Here we investigated to what extent two product peptides (one containing Q and one containing E, but otherwise identical in sequence) ionise in unseparated mixtures, in various ratios. Stapels and Barofsky^26^ analysed a large number of peptides using both ESI and MALDI and found that there were groups of peptides that were only observed when analysed using one or the other ionisation technique. They observed that properties such as the isoelectric point of the peptide made little difference in the peptides' behaviour using the two ionisation methods. Among the peptides analysed by Stapels and Barosfsky,^26^ peptides containing glutamic acid were found to have statistically significant differences in ionisation behaviour between ionisation sources and the number of times they were observed, with peptides containing glutamic acid being preferentially ionised by ESI. In addition, it has also been reported that asparagine and aspartic acid containing peptides ionise similarly under ESI conditions.^27^, ^28^ The product peptides analysed in this study showed ionisation bias when analysed using MALDI, with product peptide E consistently underestimated. Analysis of the same mixtures however using ESI showed no ionisation bias between the two peptide products. When using ESI the full range of product peptide mixtures studied was measured accurately. This accuracy was maintained when the mixtures were spiked into protein digests of small (CytC), medium (BSA) and large (collagen) proteins, at different concentrations.

There are a few possible reasons for the observed ionisation bias. It could be the way that the peptides are incorporated into the matrix, or how they behave in the plume on desorption. When analysed using LC/MS, the two peptide forms did resolve chromatographically; however, in biological mixtures such as protein digests there may be coelution of other Q‐ and E‐containing peptides. If possible, chromatographic separation of Q‐ and E‐containing peptides prior to MS analysis could help to negate potential ionisation bias.

Although this study originated with questions raised over the wide adoption of MALDI rather than ESI for analysis of ancient protein samples, the results are of much broader applicability and contribute to the body of information on peptide ionisation bias that is relevant to all those working in peptide mass spectrometric analysis, since sample handling procedures can be a cause of deamidation (e.g. protocols used in the preparation of samples for proteomic analysis, or during production of biotherapeutics). Demonstrating that ESI is much better able to generate reliable response ratios of deamidated to undeamidated peptides than MALDI is an important observation, reaching well beyond the ancient protein community.

## 5 CONCLUSIONS

Two peptides, differing only in that one contains an internal Q residue and the other an E at the same position, were synthesised in high purity using solid‐phase synthesis. The synthesis process was very successful and yielded peptide products that were significantly purer than many commercially available ‘authentic standards’.

It appears that, when choosing an ionisation source, ESI is more suitable than MALDI for accurately measuring ratios of Q‐ and E‐containing peptides. We have shown that use of MALDI results in an under‐representation of product peptide E across the full range of different percentage ratios. The level of underestimation of product peptide E varied across different peptide ratios, so that use of a simple correction factor is not sufficient to mitigate this. It should be noted that the effects of ionisation have only been studied here on one peptide sequence, and that the amino acid sequence may also have an effect on ionisation bias. However, it has been shown here that even using mixtures of synthetic products containing essentially just two peptides (product peptides Q and E), preferential ionisation of product peptide Q occurs when using MALDI. This observation is also valid in more complex peptide mixtures, such as a digest of a single protein. This observation alone is sufficient to question continued automatic use of MALDI in preference to ESI for such analyses. Our preliminary observations make it clear that further investigation is required, not only to look at different peptide sequences, but also to investigate the suitability of ESI when looking at Q to E ratios in other complex biological mixtures such as digests of protein mixtures as well as other biological matrices such as tissue samples used for imaging.

## Supporting information

Data S1. Figure S1: Multi‐step gradient showing ratios of mobile phases A and B over the course of the HPLC analysis
Figure S2: Chromatogram obtained from LC analysis of product peptide Q. The main peak is observed at tR 3.9, with two smaller peaks at tR 41 and tR 4.4 minutes. Region around the peaks enlarged in lower chromatogram.
Figure S3: MS data obtained for three peaks present in the UV chromatogram of product peptide Q. The three spectra shown correspond to peaks at tR 3.9 minutes (peak A), tR 4.1(Peak B) and tR 4.4 (Peak C).
Figure S4: UV chromatogram obtained from LC analysis of peptide E. The main peak is observed at tR 4.2 minutes, with two smaller peaks at tR 3.9 minutes and tR 4.8 minutes. Region around the peaks enlarged in lower chromatogram.
Figure S5: MS data obtained for three peaks present in the UV chromatogram of product peptide E. The three spectra shown correspond to tR 3.9 minutes (peak A), tR 4.2 (Peak B) and tR 4.8 (Peak C).
