# Supplementary material for: Ionisation bias undermines the use of matrix‐assisted laser desorption/ionisation for estimating peptide deamidation: Synthetic peptide studies demonstrate electrospray ionisation gives more reliable response ratios
Source: Rapid Commun Mass Spectrom. 2019 May 10;33(12):1049–57. doi: 10.1002/rcm.8441 (PMC6594239; doi:10.1002/rcm.8441)
Supplement: Supplementary file 1 — Data S1. Figure S1: Multi‐step gradient showing ratios of mobile phases A and B over the course of the HPLC analysis Figure S2: Chromatogram obtained from LC analysis of product peptide Q. The main peak is observed at tR 3.9, with two smaller peaks at tR 41 and tR 4.4 minutes. Region around the peaks enlarged in lower chromatogram. Figure S3: MS data obtained for three peaks present in the UV chromatogram of product peptide Q. The three spectra shown correspond to peaks at tR 3.9 minutes (peak A), tR 4.1(Peak B) and tR 4.4 (Peak C). Figure S4: UV chromatogram obtained from LC analysis of peptide E. The main peak is observed at tR 4.2 minutes, with two smaller peaks at tR 3.9 minutes and tR 4.8 minutes. Region around the peaks enlarged in lower chromatogram. Figure S5: MS data obtained for three peaks present in the UV chromatogram of product peptide E. The three spectra shown correspond to tR 3.9 minutes (peak A), tR 4.2 (Peak B) and tR 4.8 (Peak C). [file RCM-33-1049-s001.docx]

**Supplementary note 1 (S1) - peptide synthesis methodology**

**Reagents**

Throughout the following protocols, purified water refers to water purified using a MilliQ system, having a resistivity of 18 MΩ. Amino acids and reagents used were purchased from Sigma Aldrich. These included: pre-loaded glycine resin (H-Gly-2-CITrt resin), Fmoc-Val-OH, Fmoc-Glu(tBu)-OH,Fmoc-Gln(tBu)-OH, Fmoc-Gly-OH, Fmoc-Hyp(tBu)-OH, Fmoc-Tyr(tBu)-OH, Fmoc-Ala-OH. Fritted polypropylene (PP) tubes (10 mL) were used as vessels for the solid phase reactions. During the reaction, samples were agitated using a Stuart blood rotator. Reagents used included: trifluoroacetic acid (TFA), *N,N*-dimethyl formamide (DMF), dichloromethane (DCM), methanol (MeOH), and piperidine (PIP), triisopropylsilane (TIS), (6-chloro-1H-benzotriazol-1-yl)oxy](dimethylamino)-*N,N*-dimethylmethaniminium hexafluorophosphate (HCTU), *N,N*-diisopropylethylamine (DIPEA), purified water, cold diethyl ether, liquid nitrogen.

**Coupling reaction**

The product Q- and E-containing peptides were prepared at the same time in two separate PP tubes. To each tube a 100 mg aliquot of H-Gly-2-CITrt resin (loading ratio, 1.1 mmol/g) plus 3 mL of DMF was added, to swell the resin. The tubes were rotated on the Stuart rotator for 30 minutes at room temperature. The DMF was then evacuated and this process was repeated three times. Fmoc-Val-OH (187 mg) and HCTU (223 mg) were dissolved in 3 mL of DMF, and 203 µL DIPEA was added. This solution was prepared in duplicate with 3 mL of the solution added to each of the PP tubes. The tubes were then transferred to the Stuart rotator and left to rotate for one hour. The solution was evacuated from both tubes and Fmoc deprotection was carried out by adding 3 mL of piperidine solution (20 % in DMF) to each tube and rotating for two minutes. The piperidine solution was then evacuated and this process was repeated a further four times. Following Fmoc deprotection, 3 mL of DMF was added to each tube and the sample was left to rotate for 2 minutes. The DMF was then evacuated and this process was repeated a further four times.

The coupling reaction was repeated with the same conditions, substituting valine for the following amino acid derivatives and amounts: Fmoc-Glu(tBu)-OH (234 mg)/ Fmoc-Gln(tBu)-OH 234 mg), Fmoc-Gly-OH (163 mg), Fmoc-Hyp(tBu)-OH (225 mg), Fmoc-Gly-OH (163 mg), Fmoc-Tyr(tBu)-OH (253 mg), Fmoc-Ala-OH (171 mg), Fmoc-Tyr(tBu)-OH (152 mg). Each amino acid was coupled once, except for the Gly preceding Hyp in the peptide sequence, for which the coupling step was performed twice.

Cleavage and isolation of the peptides

The resin was isolated by filtration and dried under high vacuum for three hours. The cleavage solution consisted of H_2_O:TIS:TFA (2.5:2.5:95, v:v:v). 3 mL of cleavage solution was added to each tube containing the resin. The tubes were left to rotate for two hours. The sample solution was then separated from the resin by filtration. The resin was then washed twice with 3 mL of pure (≥ 99.0 %) TFA. The two washes were then combined with each of the corresponding sample solutions. 40 mL of ice cold diethylether was transferred into PP falcon tubes and left to chill at approx. -5 °C for 2 hours. The sample solutions were each transferred into separate falcon tubes of cold diethyl ether, in which the product precipitated. Each sample was centrifuged for ten minutes (4,000 x g) and the supernatant removed and discarded. The pellet was then re-suspended in ice-cold diethylether and the process repeated a further three times. The remaining pellet was dissolved in 2 mL of water and lyophilised. The resulting peptide samples were stored in the freezer (approx. -20 °C).

**Supplementary note 2 (S2)- Analysis of peptide purity by LC-MS**

The lyophilised peptides were suspended in water at a concentration of 100 ppm. Each peptide was analysed using an HPLC-HCTultra PTM Discovery System (Bruker Daltonics), fitted with a symmetry C18 3.5 µm (4.6 x 7.5 mm) column (Waters) using mobile phases of acetonitrile (A) and water (B). The elution was isocratic with a flow rate of 1 mL/min and a total run time of 9 minutes. The multi-step gradient of A and B over the course of the run is summarised in Table 1/ Figure 1.

Table 1

| Time (minutes) | % A |
| --- | --- |
| 0 | 95 |
| 1 | 95 |
| 6 | 70 |
| 6.1 | 5 |
| 7 | 5 |
| 7.2 | 95 |
| 9 | 95 |


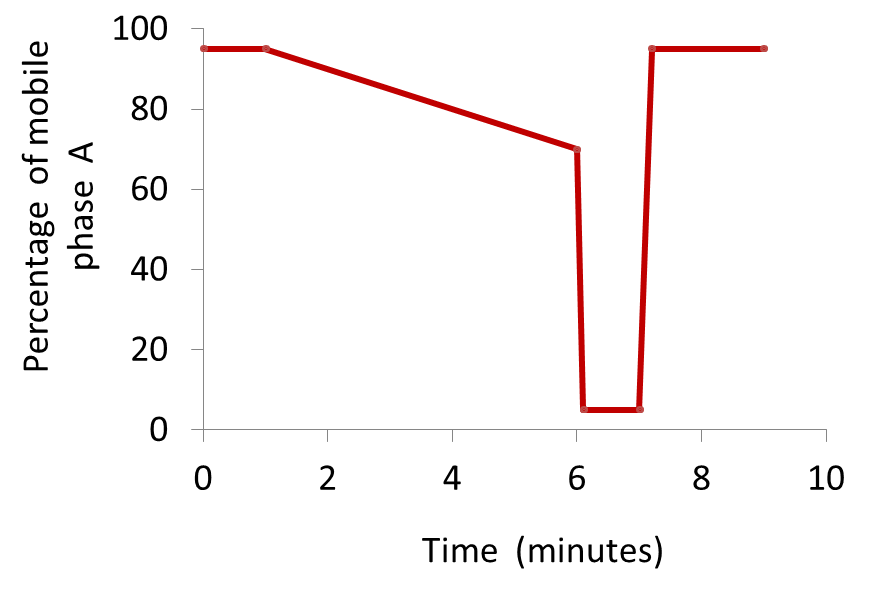


Figure 1: Multi-step gradient showing ratios of mobile phases A and B over the course of the HPLC analysis

Purity analysis of peptides by LC-MS and MALDI-TOF-MS

Demonstration of peptide sequences

Product ion analysis of the spots prepared with either 100 % product peptide E or product peptide Q was carried out on the MALDI-TOF/TOF instrument. The assignment of the sequence of each of the peptides was made on interpretation of the product ion spectra. The product ion spectrum for product peptide E showed a mixture of both the Q- and E-containing peptides (the precursor selection window on the instrument used is >1 *m/z* unit). Harsh acidic treatments were used to cleave the synthetic peptides from the resin; it is therefore possible that some of the glutamine in the product peptide Q may have undergone minor deamidation during synthesis (Simpson et al., 2016).

**Analysis of the purity of product peptide Q**

To assess the purity of the two synthesised peptides, each peptide was first analysed separately by reversed phase LC-ESI-MS. UV absorbance (210 – 380 nm) data were also collected. As expected, similar absorbance patterns were observed for each of the two peptides. The UV chromatogram for product peptide Q contained three peaks. The main peak was at *t*_R_ 3.9 minutes and had a short tail. Two smaller peaks at *t*_R_ 4.1 _and_ *t*_R_ 4.4 were also detected (Figure 2).


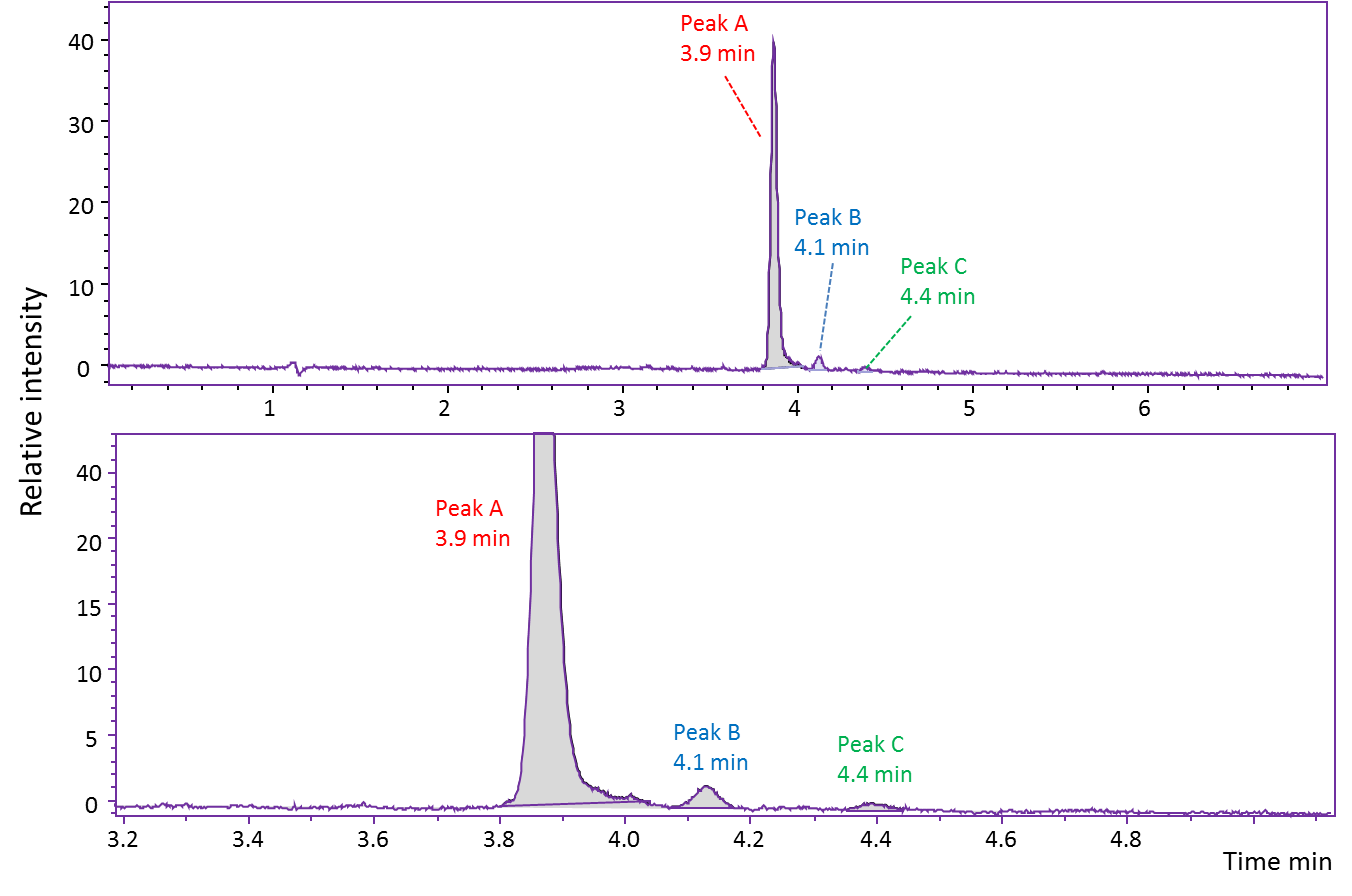


Figure 2: Chromatogram obtained from LC analysis of product peptide Q. The main peak is observed at *t*_R_ 3.9, with two smaller peaks at *t*_R_ 41 and *t*_R_ 4.4 minutes. Region around the peaks enlarged in lower chromatogram.

The spectrum generated from the main peak at *t*_R_ 3.9 min contained a number of singly and doubly charged signals (Figure 3, peak A). Two peaks in the spectrum corresponded to the mass expected for peptide Q: M+H^+^ at *m/z* 927.4 and [M+2H]^2+^ at *m/z* 464.3. In addition to the main peak observed in the chromatogram, there was a second peak observed at *t*_R_ 4.1 min (Figure 4, Peak B). ESI-MS analysis of this peak yielded signals corresponding to the expected peptide E, with M+H^+^ at *m/z* 928.3 and [M+2H]^2+^ at *m/z* 464.7. A third peak was observed at *t*_R_ 4.4 minutes (Figure 4). ESI-MS analysis of this peak yielded a spectrum containing one doubly charged signal at *m/z* 422.6 and singly-charged signals at *m/z* 448.8 and *m/z* 806.3 (Figure 3, Peak C). Attempts to identify these minor species using product ion analysis failed due to the low signal intensities and so the components in this minor peak remain unassigned.


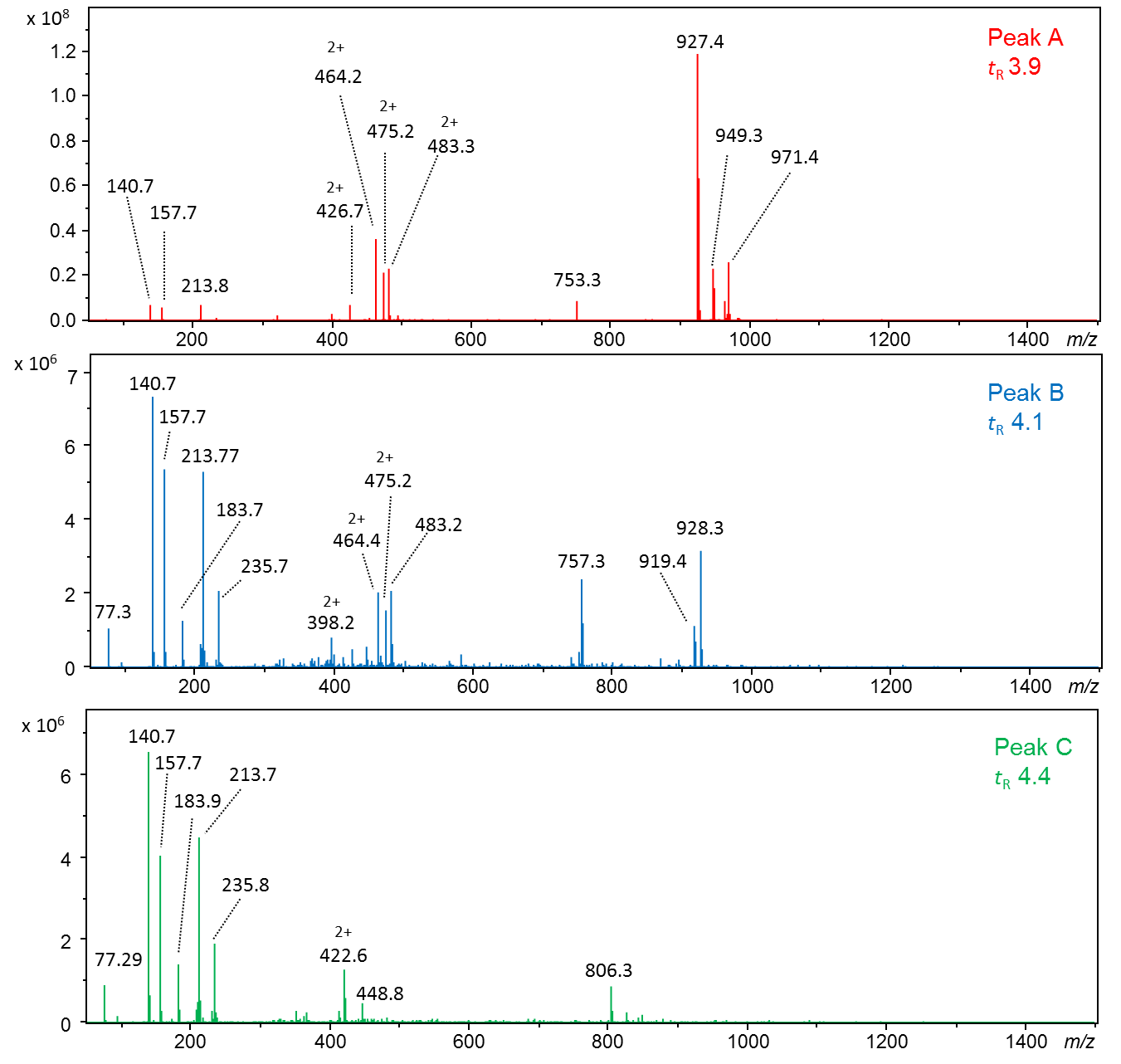


Figure 3: MS data obtained for three peaks present in the UV chromatogram of product peptide Q. The three spectra shown correspond to peaks at *t*_R_ 3.9 minutes (peak A), *t*_R_ 4.1(Peak B) and *t*_R_ 4.4 (Peak C).

On the basis of the peak areas in the UV chromatogram, product peptide Q is estimated to be ~ 94.81 % pure and to contain 3.76 % of peptide E, assuming that all components give a similar UV response. From this analysis it is not possible to distinguish whether the small amount of peptide E in product peptide Q arises from deamidation during peptide synthesis, or from the presence of glutamic acid with the glutamine precursor used to make the peptide.

**Analysis of the purity of product peptide E**

The UV chromatogram obtained for product peptide E contained one main peak with a *t*_R_ of 4.2 minutes and two smaller peaks at *t*_R_ 3.9 and *t*_R_ of 4.8 (Figure 4).


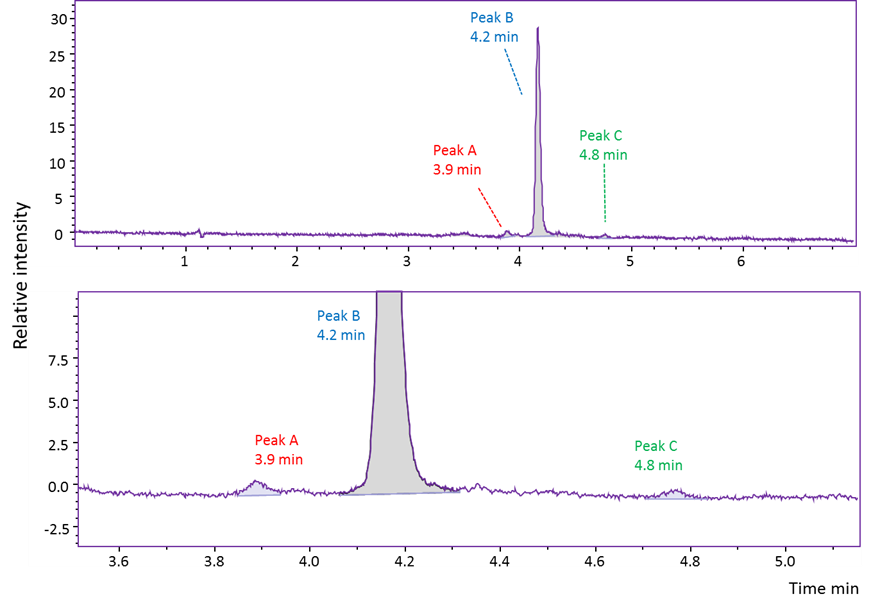


Figure 4: UV chromatogram obtained from LC analysis of peptide E. The main peak is observed at *t*_R_ 4.2 minutes, with two smaller peaks at *t*_R_ 3.9 minutes and *t*_R_ 4.8 minutes. Region around the peaks enlarged in lower chromatogram.

ESI-MS analysis of the peak at *t*_R_ 4.2 resulted in signals at *m/z* 464.7 ([M+2H]^2+^) and *m/z* 928.4 (M+H^+^) (Figure 5) for the expected peptide. ESI-MS analysis of the peak with a *t*_R_ of 3.9 minutes resulted in signals at *m/z* 927.4 and 464.2, corresponding to M+H^+^ and [M+2H]^2+^ for the product peptide Q. This conclusion is consistent with the results of product ion analysis of the peptide E product, which was demonstrated to be a mixture of the E- and the Q-containing peptides. The signals in the mass spectrum of the peak in the chromatogram at *t*_R_ of 4.8 minutes (Figure 5) are 1 amu higher than those in the peak at *t*_R_ 4.4 minutes from the product peptide Q analysis, and are therefore consistent with the presence of E rather than Q in these components. Taking into account the two impurity peaks, product peptide E was estimated to be ~ 95.40 % pure and to contain ~2.68 % of product peptide Q


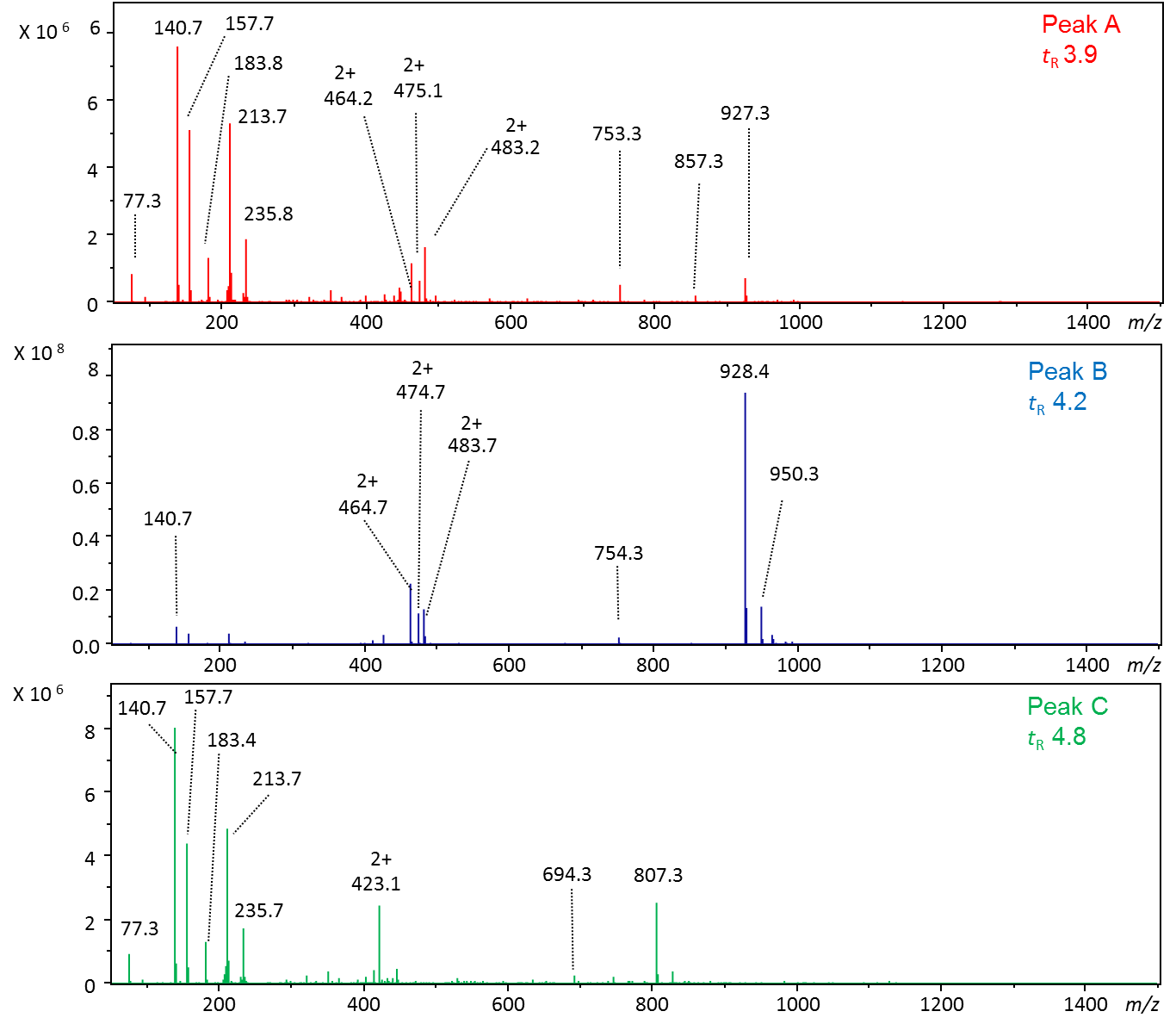


Figure 5: MS data obtained for three peaks present in the UV chromatogram of product peptide E. The three spectra shown correspond to *t*_R_ 3.9 minutes (peak A), *t*_R_ 4.2 (Peak B) and *t*_R_ 4.8 (Peak C).
